# Differences in Histopathology and Local Immune Response in Steady and Progressive Natural Transmissible Venereal Tumors in Mexican Dogs

**DOI:** 10.3390/ani16081262

**Published:** 2026-04-20

**Authors:** Ileana Zorhaya Martínez-Ramos, Diego Pérez-Maroto, Natalia García-Álvarez, Patricia Barroso, Adan García Balbuena, Guadalupe Núñez-Martínez, María Benedicta Bottini Luzardo, Juan Francisco García Marín, Ana Balseiro

**Affiliations:** 1 Facultad de Medicina Veterinaria y Zootecnia, Universidad Popular Autónoma del Estado de Puebla, Puebla 72410, Mexico; ileanazorhaya.martinez@upaep.mx (I.Z.M.-R.); adan.garcia@upaep.mx (A.G.B.); guadalupe.nunez@upaep.mx (G.N.-M.); 2 Secretaría de Ciencia, Humanidades, Tecnología e Innovación del Estado de Puebla, Puebla 72400, Mexico; 3 Departamento de Sanidad Animal, Facultad de Veterinaria, Universidad de León, 24071 León, Spain; dperem10@estudiantes.unileon.es (D.P.-M.); ngara@unileon.es (N.G.-Á.); pbars@unileon.es (P.B.); jfgarm@unileon.es (J.F.G.M.); 4 Facultad de Medicina Veterinaria y Zootecnia No. 2. Universidad Autónoma de Guerrero, Cuajinicuilapa 41940, Mexico; 17906@uagro.mx; 5 Departamento de Sanidad Animal, Instituto de Ganadería de Montaña (IGM-CSIC), 24346 León, Spain

**Keywords:** TVT, immunohistochemistry, macrophages, B lymphocytes, dogs, morphological types

## Abstract

The canine transmissible venereal tumor (TVT) is a neoplasm of the external genitalia of dogs, considered one of four reported contagious tumors in animals. These tumors have different presentations, with steady, progressive, or regressive stages. In some areas of Mexico, where the prevalence of TVT is high (5.15%), two morphological types are usually observed: one steady, pedunculated, strawberry-like (Type A) and one progressive, multilobulated, cauliflower-like (Type B). This study aimed to characterize the histopathological and inflammatory infiltrate patterns in eight stray dogs showing both morphological types of natural TVT (n = 4 each), to identify potential differences between tumor morphologies. Histopathological and immunohistochemical techniques were applied to tumor samples to evaluate the interaction between pathological morphology and the following cell markers: ionized calcium-binding adaptor molecule 1 (IBA1) for activated macrophages (including resident macrophages), inducible nitric oxide synthase (iNOS) for M1 macrophages, CD163 for M2 macrophages, CD3 for T lymphocytes, CD20 for B lymphocytes, and lambda light chain for plasma cells. The results showed a greater inflammatory infiltrate in Type A tumors than in Type B ones, with a parallel increase in activated macrophages and B lymphocytes. The presence of M1 and M2 macrophages was scarce in both types of tumors, and T lymphocytes were almost absent. This study reveals a stronger and more balanced local immune response in dogs with Type A TVTs compared with Type B tumors, which may underlie differences in tumor characteristics, although individual tumor heterogeneity should be considered.

## Simple Summary

The canine transmissible venereal tumor (TVT) is a neoplasm of the external genitalia of dogs, considered one of four reported contagious tumors in animals. This study aimed to characterize the histopathological and inflammatory infiltrate patterns in eight stray Mexican dogs showing two pathological morphologies of natural TVT (n = 4 each), to identify potential differences between tumor morphologies. The presence of M1 and M2 macrophages was scarce in both types of tumors, and T lymphocytes were almost absent. This study reveals a stronger and more balanced local immune response in dogs with Type A TVTs compared with Type B tumors, which may underlie differences in tumor characteristics, although individual tumor heterogeneity should be considered.

## 1. Introduction

The canine transmissible venereal tumor (TVT), also called the Sticker tumor, is a neoplasm of the external genitalia of dogs, which commonly affects both sexes. It is one of four reported contagious tumors in animals [1,2], where the infecting cells are transplanted by direct contact and grow similarly to a graft [3]. TVT is considered the oldest neoplasm in the world, originating from an individual 11,000 years ago [2]. Indeed, molecular studies have demonstrated that TVT isolates from diverse geographic regions and time periods are clonal in nature, despite two subtypes being recognized. The tumor remains a very common canine infectious disease distributed worldwide, mostly linked to national development status and access to veterinary care [4]. In this line, higher prevalences have been observed in tropical and subtropical areas, where stray and wild dogs exhibit unrestrained sexual activity, compared with regions where canine populations are controlled, such as North–Central Europe and North America [1,3]; therefore, areas with higher rates of neutering also tend to have a lower incidence of the disease [4]. In addition, significant temporal fluctuations in prevalence occur within endemic zones [4].

The tumor has different presentations (steady, progressive, or regressive) and its gross morphology may be cauliflower-like, pedunculated, nodular, papillary, or multilobulated, ranging from a small nodule of 5 mm in diameter to a large mass measuring more than 10 cm [1]. The neoplasm is firm though friable, with the superficial part commonly ulcerated, inflamed, and secondarily infected. In females, the tumor can appear as an irregular, ulcerated, and friable mass within the vagina, protruding from the vulva. In males, the penis or prepuce may present TVT with variable forms and consistencies, i.e., single or multiple, soft or firm [5]. In some cases, the neoplasm can be found in skin locations through bites or wounds [6], and it can also metastasize to regional lymph nodes, ovaries, and other organs, although metastases to internal viscera are rare [3,7]. Buccal and ocular lesions have also been described [8,9].

Histologically, the tumor exhibits variations depending on its stage of growth or regression. In the early growth phase, cells are round, oval, or polyhedral with pale cytoplasm, sometimes vacuolated, and the nuclei are large and contain prominent nucleoli [3]. Cell size variability and frequent mitotic figures are common. As the tumor mass increases in size, the cells become tightly packed and irregularly ovoid, and fibroblast-like cells appear, possibly indicating a transformation of the tumor cells. Many lymphocytes, a few plasma cells, and occasional macrophages can be scattered throughout the tumor [10]. Specifically, higher mast cell and microvessel counts have been shown in progressing tumors at invasive edges, suggesting mast cells promote TVT progression via vascularization, which may serve as a histological marker of tumor stage [11].

In the absence of an available vaccine, there are multiple treatment approaches, including surgical intervention, radiotherapy, immunotherapy [12,13], and chemotherapy [14,15,16], with vincristine as one of the most successful chemotherapeutic agents [17]. However, spontaneous regression can also occur after months of rapid progression, and dogs may develop immunity and resistance [18]. Stromal cells and extracellular matrix are important in the progression or regression of TVT. For example, as the tumor regresses, the number of intratumoral lymphocytes, mostly T cells, as well as the expression of α-smooth muscle actin (α-SMA), increase [19]. On the other hand, TVT cells can evade immune detection by secreting inhibitory cytokines or downregulating immune cells [20,21,22]. In this line, during the initial proliferative phase, there is minimal expression of major histocompatibility complex (MHC) antigens, which markedly increase after a few weeks due to lymphocyte infiltration, favoring tumor regression [5].

In different areas of Mexico, where the prevalence of TVT is high (5.15%) [23], two predominant morphological presentations have been identified, leading to differences in the clinical and therapeutic approach. The first type is pedunculated, resembling a strawberry shape, while the second is multilobulated, with deep infiltration into the mucosa displaying a cauliflower-like appearance. This study aimed to characterize the histopathological and inflammatory infiltrate patterns of both TVT types to identify potential differences between tumor morphologies to likely establish a more effective treatment strategy in affected dogs.

## 2. Materials and Methods

### 2.1. Animals and Sampling

A total of 64 stray dogs were diagnosed with TVT in Puebla, Guerrero, and Mexico City (Mexico) in 2024. Of these, eight female dogs of different breeds (i.e., five mixed-breeds, two Belgian Shepherds, and one Pit Bull) and ages (from two to seven years) were included in this study (Table 1).

**Figure 1:**
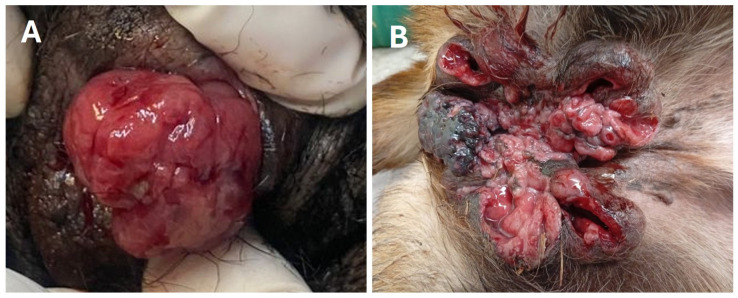
Gross appearance of transmissible venereal tumors (TVT) in female stray dogs showing strawberry-like Type A (**A**) and cauliflower-like Type B (**B**) morphological types in the vagina. The sizes of the tumors are 2 × 2 cm (**A**) and 10 × 10 cm (**B**).

All animals showed naturally occurring TVT restricted to the external genital membranes and were classified into two groups according to the morphology and stage of growth: four were pedunculated strawberry-like (Type A) and measured between 0.5 and 5 cm in diameter, and four were multilobulated cauliflower-like with mucosal infiltration (Type B) and size between 5 and 10 cm in diameter (Figure 1). This morphological variation directly influences the choice and efficacy of treatment, i.e., while Type A is easier to manage, Type B requires a more complex and expensive therapeutic approach and implies a higher risk of recurrence.

The dogs were captured for routine veterinary clinical procedures (i.e., vitamin administration, sterilization, deworming, and surgical removal of tumors). Based on the tumor gross morphology, Type A tumors were easily and completely removed by excisional biopsy, as they do not exhibit an infiltrative pattern. In contrast, incisional biopsies were performed in Type B cases, as the tumor infiltrates the mucosa. Tumor samples were subsequently fixed in 10% neutral buffered formalin for further histopathological evaluation and diagnosis. Ethical committee approval was not required, as the samples were obtained during routine veterinary clinical practices officially carried out in Mexico in stray dogs.

### 2.2. Histopathology and Immunohistochemistry

Samples for histopathology were routinely processed and embedded in paraffin wax. Semi-serial 3 µm sections were cut, mounted on glass microscope slides, and stained with hematoxylin and eosin and Masson’s trichrome stain for specific visualization of the connective tissue.

For the immunohistochemical study, serial paraffin-embedded sections (3 μm) were used. Immunohistochemistry consisted of the detection of six cell populations using the following primary antibodies: ionized calcium-binding adaptor molecule 1 (IBA1) for activated macrophages (including resident macrophages), inducible nitric oxide synthase (iNOS) for M1 macrophages (pro-inflammatory and involved in host defense), CD163 for M2 macrophages (anti-inflammatory and involved in tissue repair and healing), CD3 for T lymphocytes, CD20 for B lymphocytes and lambda light chain for plasma cells. After deparaffinization, antigen retrieval was carried out using sodium citrate buffer (10 mmol/L, pH 6.0) with heat induction by microwave for 20 min. Endogenous peroxidase activity was subsequently blocked by incubation in a hydrogen peroxide (0.5%) solution in distilled water for 30 min at room temperature. The tissue sections were then incubated overnight at 4 °C in a humidified chamber with commercial monoclonal or polyclonal antibodies diluted in Tris-buffered saline with bovine serum albumin (TBS+BSA) 0.1% (Table 2). Afterwards, they were washed with TBS 1x, and incubated with a secondary antibody (Vector Laboratories, Newark, CA, USA), diluted 1:200 in TBS+BSA 0.1% (Table 1), followed by incubation with the conjugate avidin–biotin peroxidase complex (ABC Standard, Vector Laboratories, Newark, CA, USA) in TBS 1x for 30 min. Labeling was visualized using the Vector^®^ NovaRed™ peroxidase substrate kit (Vector Laboratories, Newark, CA, USA) as a chromogen substrate. Finally, the slides were counterstained with Mayer’s hematoxylin, dehydrated, and mounted with DPX (Fluka, Sigma, St. Louis, MO, USA). Negative and positive controls were used in each protocol. The negative control consisted of an additional slide without the primary antibody, and the positive control was a lymph node from a road-killed badger.

To distinguish the primary antitumor immune response from secondary inflammation due to bacterial infection, two criteria were applied: (i) cellular localization and pattern: inflammatory infiltrate directly associated with tumor tissue was identified in histological samples, consistent with a primary immune response, while the presence of neutrophils and superficial cellular debris was interpreted as secondary inflammation due to bacterial infection; and (ii) lesion morphology: ulcerated tumors exhibited areas of necrosis and superficial bacterial colonization, whereas the immune response against the tumor was predominantly observed within the deeper tumor stroma.

### 2.3. Evaluation and Quantification of Cell Types

Immunostained tissue slides were scanned at the Microscopy Service of the University of León using an Olympus BX51 microscope (Olympus, Tokyo, Japan), equipped with an Olympus XC10 camera (Olympus, Tokyo, Japan). The digitally scanned samples were then analyzed using QuPath v. 0.5.1 image analysis software (QuPath, University of Edinburgh, Edinburgh, Scotland).

To quantify immunopositive cells, five randomly selected fields at 4× magnification were analyzed for each sample and antibody, resulting in a total of 240 fields. For each sample and antibody, cell counting was performed over a total area of 10,392,330.3 µm^2^. All cell counts were conducted in a blinded manner, without prior knowledge of the tumor type present in the different samples.

### 2.4. Statistical Analysis

A generalized linear mixed model was carried out to assess the differences in the scores of cell markers between tumor morphopathologies. The response variable was the score values (i.e., the percentage of immunopositive cells measured in each of the five randomly selected fields per dog; n = 240). In this model, tumor morphopathology (strawberry-like Type A or cauliflower-like Type B), cell markers (IBA1-macrophages, iNOS-M1 macrophages, CD163-M2 macrophages, CD3-T lymphocytes, CD20-B lymphocytes, and lambda light chain-plasma cells), and their interaction were introduced as fixed explanatory variables. The individual dog was included as a random effect to account for repeated measures. This approach allowed the separation of within-animal variability from effects due to tumor type and cell marker. Significant differences found among groups were explored through multiple pairwise comparison post-hoc analyses with the Tukey test. Statistical significance was accepted at *p* < 0.05. The model was performed using the lme4 package in R software, version 4.0.2 [24].

## 3. Results

### 3.1. Histopathological and Inflammatory Infiltrate Pattern

**Figure 2:**
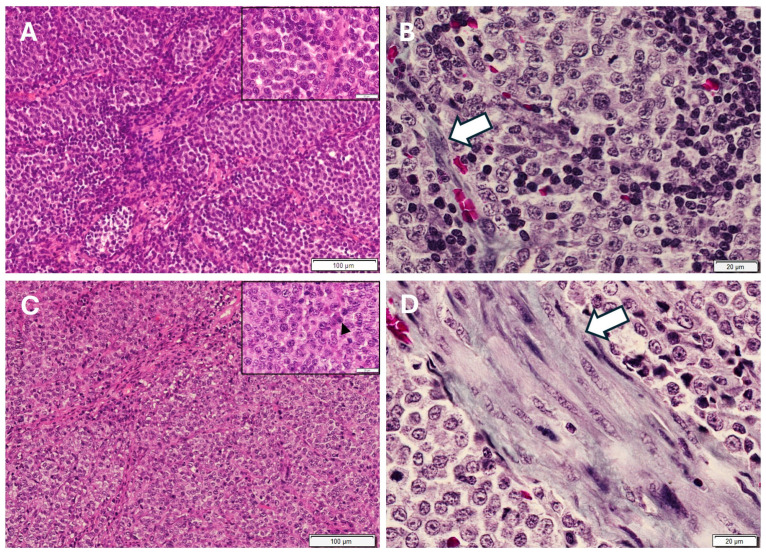
Microphotographs of strawberry-like Type A (**A**,**B**) and cauliflower-like Type B (**C**,**D**) transmissible venereal tumors in dogs. Neoplastic cells are round (insets), and mitotic figures (arrowhead) are observed (inset (**C**)). Note the higher inflammatory infiltrate in Type A tumors, forming cords or clusters of cells (**A**). Stroma (blue-stained) is minimal in both types of tumors ((**B**,**D**), white arrows), although slightly higher in Type B tumors. Hematoxylin and eosin (**A**,**C**) and Masson’s trichrome stain (**B**,**D**).

**Figure 3:**
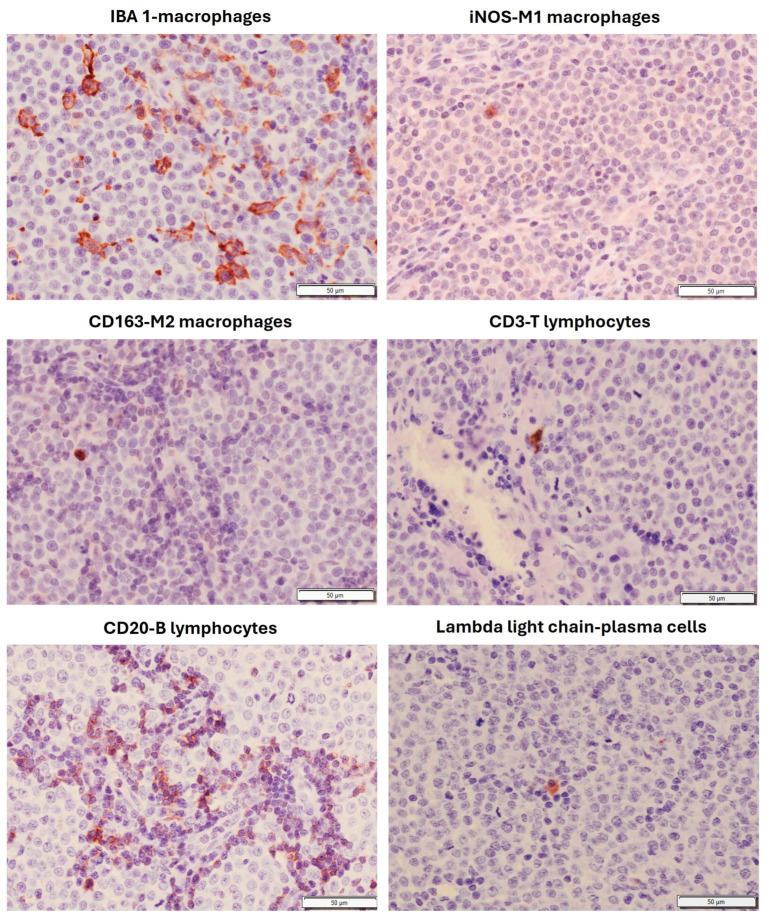
Local inflammatory infiltrate in a naturally transmissible venereal tumor in dog showing strawberry-like Type A morphology. Six primary antibodies were used. Note the intense inflammatory infiltrate in which activated macrophages and B lymphocytes predominate. Immunohistochemical technique: avidin–biotin complex.

**Figure 4:**
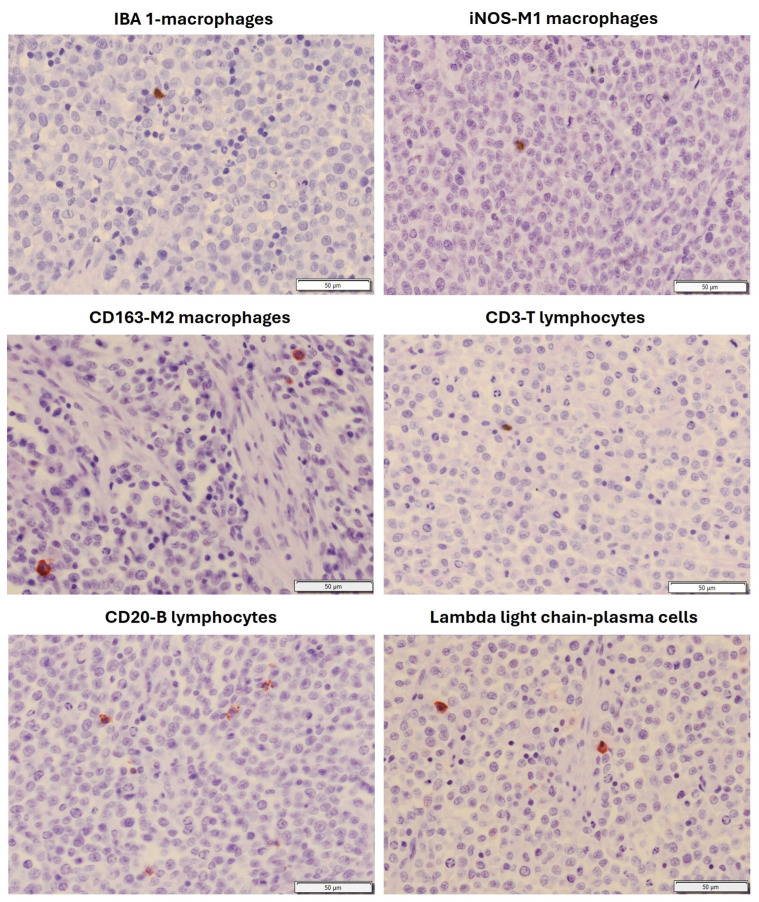
Local inflammatory infiltrate in a naturally transmissible venereal tumor in dog showing cauliflower-like Type B morphology. Six primary antibodies were used. Note the low inflammatory infiltrate in which B lymphocytes and plasma cells slightly predominate. Immunohistochemical technique: avidin–biotin complex.

In both types of tumors, neoplastic cells were organized in compact masses or sheets within a minimal stroma, which was slightly higher in Type B TVT (Figure 2). The cells were round, ovoid, or polyhedral (Figure 2). They had a moderate amount of eosinophilic cytoplasm and large, round, hyperchromatic nuclei, with distinctly marginal chromatin and large central nucleoli. Many mitotic figures were found (six to eight per 400x field), and hemorrhages, small multifocal necrosis areas, and vascular neoformation were also common features within the tumors. An inflammatory infiltrate, mainly consisting of activated macrophages and B lymphocytes and fewer plasma cells, was also observed in both types of tumors (Figure 3 and Figure 4), but the number and type of infiltrating inflammatory cells depended on the tumor morphology, Type A or Type B (see Section 3.2). In Type A tumors, the inflammatory infiltrate was higher than in the Type B morphological type and was distributed mainly forming cords or clusters of cells between the neoplastic cells (Figure 2), whilst in the Type B tumors, the inflammatory infiltrate was mostly scattered throughout the neoplasm and surrounding it, although cords or clusters were also occasionally observed (Figure 2). The presence of M1 and M2 macrophages was scarce in both types of tumors, as well as T lymphocytes, which were almost absent (Figure 3 and Figure 4).

### 3.2. Relationship Between Tumor Morphopathology and Cell Markers

**Figure 5:**
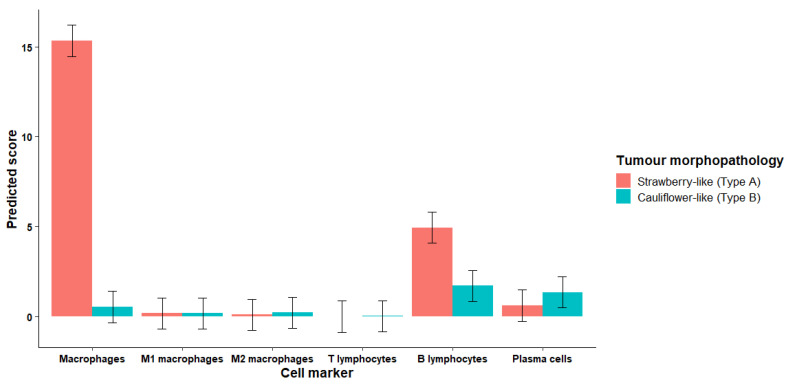
Predicted score (± confidence interval 95%) for six cell markers (IBA1-macrophages, iNOS-M1 macrophages, CD163-M2 macrophages, CD3-T lymphocytes, CD20-B lymphocytes, and lambda light chain-plasma cells) for each transmissible venereal tumor (TVT) morphopathology (strawberry-like Type A or cauliflower-like Type B). Scores were calculated from five randomly selected fields per tumor in eight dogs with TVT (four with Type A morphopathology and four with Type B morphopathology). Note that macrophages and B lymphocytes are significantly higher in Type A tumors.

The interaction between tumor morphopathology and cell markers was statistically significant. Specifically, the scores of IBA1 (activated macrophages) and CD20 (B lymphocytes) were significantly higher in Type A tumors compared with Type B tumors (*p* < 0.001 and *p* = 0.02, respectively; Table 3 and Figure 5). The scores of the remaining markers (i.e., CD3, iNOS, CD163, and lambda light chain) did not vary significantly with tumor morphology (all *p* > 0.240; Table 3). Furthermore, most of the variation in cell marker scores was observed within tumor types, at the level of individual fields (residual variance = 9.53; standard deviation (SD) = 3.09), whereas variation attributable to differences between dogs (random effect) was relatively low 1.13 (variance = 1.13; SD = 1.06).

## 4. Discussion

In this study, by comparing the histopathological and immune cell marker expression in Type A and Type B tumors, we found differences in the pathological and immunological mechanisms underlying those morphological variations. First, a greater inflammatory infiltrate was present in Type A tumors compared with Type B ones, and second, a parallel increase in activated macrophages and B lymphocytes infiltrating Type A tumors was observed. Both pathological findings might be related to the reduced tumor size in Type A TVTs. The immunosuppressive effect of the tumoral cells in this neoplasia is well known, and the downregulation of the expression of MHC genes leads to the absence of MHC molecules, especially during the progression phase [1,5]. Specifically, MHC type II is completely inhibited in the progression stage, whilst MHC type I continues to be expressed up to 10% [25]. However, during the regression phase, those molecules tend to be expressed, leading to the activation of immunological cells [26], which can favor regression. Also, the tumoral cells secrete transforming growth factor β1 (TGβ1), suppressing the immune response [1,5]. In contrast, IL-6, IL-15, and γ-interferon (γ-IFN) produced during the regression phase collaborate in restoring the expression of MHC molecules and the consequent effective immune response [21].

The higher inflammatory infiltrate in Type A and the differences in the detection of macrophages and B lymphocytes between the two pathological types may be related to differences in the stage of the neoplasia (i.e., steady or progressive) [20]. As there was greater quantification of macrophages and B lymphocytes in the Type A tumors, TVTs with this morphology might have restored the MHC expression, since both macrophages and B lymphocytes express MHC type II molecules [27], which are totally suppressed during the tumor progression phase. However, this hypothesis cannot be confirmed with the present approach, as no longitudinal follow-up of tumor evolution was performed. Further studies, including more animals and temporal monitoring of tumor progression and regression, are needed to elucidate the dynamics of MHC expression and immune cell infiltration in TVT. Nevertheless, it should be clarified that both tumor morphologies were observed in the animals from the outset, and that Type A tumors do not represent an early stage of Type B. On the other hand, the allograft inflammatory factor 1 (AIF-1) is considered homologous to the IBA1 molecule [28]. Several studies in human cancers have associated AIF-1 upregulation with malignant characteristics, e.g., in glioma, hemangioma, breast cancer, and hepatocellular carcinoma [28,29]. However, in this study, a very high expression of IBA1 was detected in steady Type A TVTs, which are likely to exhibit lower probabilities of growth. Furthermore, although no significant differences were observed between M1 and M2 macrophages and their overall scores were low in the eight dogs studied, it is well known that they have different roles based on their activation state, i.e., while M1 macrophages are involved in tumor suppression, M2 macrophages are associated with tumor cell proliferation [30]. In this regard, Type A and Type B TVTs exhibited distinct tissue microenvironments. Type A presented a remodeled stroma with numerous macrophages (high IBA1 expression), suggesting organized growth and either steady behavior or potential for immune activation at later stages. In contrast, Type B, with very low IBA1 expression, demonstrated immunosilent and more aggressive characteristics, with minimal macrophage recruitment and invasion into adjacent tissues. As previously mentioned, both tumors exhibited comparable levels of M1 (pro-inflammatory) and M2 (anti-inflammatory) macrophages, despite differences in overall infiltration and tissue composition. All these findings might suggest different stages of TVT progression, although specific characteristics on histopathology, infiltration immune pattern, and invasive behavior also support the possibility of two distinct subtypes of TVT with specific stroma and immune environment.

The regression phase of this neoplasia is typically characterized by a high infiltration of T lymphocytes [26]. Although in this study, T lymphocyte detection was very low and did not differ between tumor types, other studies have described a higher presence of CD3+ T lymphocytes in TVT samples with a stable or decreasing size [31]. In this line, based on the main immune cell present, TVT has been classified into three cytomorphological patterns, –plasmacytoid, lymphocytoid, and mixed–, which differ in their biological behavior, interaction with the immune system, and response to treatment [32,33]. These phenotypic differences are thought to reflect distinct underlying molecular programs that shape tumor progression and therapeutic outcomes. For example, plasmacytoid tumors are frequently associated with higher mitotic activity and decreased sensitivity to chemotherapy, partly due to overexpression of the MDR1 gene encoding P-glycoprotein [34]. In contrast, lymphocytoid tumors tend to exhibit lower proliferative indices and greater chemosensitivity, suggesting a more favorable prognosis [35]. The mixed subtype is considered an intermediate or transitional form, potentially reflecting a dynamic progression between less and more aggressive tumor states [36]. Recent transcriptomic analyses further support these distinctions, demonstrating that each cytomorphological subtype is driven by specific gene expression profiles [37]. Plasmacytoid tumors show strong immune-related transcriptional signatures, including pathways involved in cytokine signaling, complement activation, and MHC class II-mediated antigen presentation, consistent with active immune engagement alongside possible immune evasion mechanisms. By contrast, lymphocytoid tumors display increased expression of genes related to cell cycle regulation and proliferation, suggesting a more proliferative but relatively immunologically quiescent phenotype. Finally, mixed tumors exhibit transcriptional patterns that lie between these two extremes, reinforcing the concept of a continuum in tumor evolution.

Altogether, for an effective immune response, adequate control of the inflammatory response is necessary [38]. In this study, dogs with Type A TVTs seemed to display an effective and balanced local immune response. The macrophage activation in those animals was elevated, suggesting a robust innate immune response, while the higher levels of B lymphocytes indicate an effective humoral immune response. On the contrary, the local immune response in the Type B tumors showed lower scores of macrophages and B lymphocytes, suggesting a reduced and inefficient local immune response. However, it is important to note that certain heterogeneity exists within individual tumors at the microscopic level. This suggests that local immune activity may vary between different regions of the same tumor, which could impact the overall effectiveness of the immune response. Therefore, caution should be taken when generalizing findings based solely on tumor pathology. Future longitudinal studies are warranted to further elucidate how the immune microenvironment evolves across tumor stages and how it influences stability or spontaneous regression in TVTs.

## 5. Conclusions

This study reveals differences in tumor histopathology and local inflammatory patterns between Type A and B TVTs, with a stronger and more balanced local immune response in dogs showing Type A tumors. The study has also deepened the knowledge of histopathological characteristics of Type B TVT, which can help to understand its biological and feature characteristics, since Type B does not respond well to the available treatments.

## Figures and Tables

**Table 1:** Data on the dogs included in this study.

Dog	Breed	Sex	Age (Years)	Location in Mexico	Type of TVT ^1^
1	Pit bull	Female	7	Cuajinicuilapa, Guerrero	A ^2^
2	Mixed-breed	Female	2	Huejotzingo, Puebla	A ^2^
3	Mixed-breed	Female	3	Balcones del sur, Puebla	A ^2^
4	Mixed-breed	Female	4	Amozoc, Puebla	A ^2^
5	Belgian Sheperd	Female	6	Huejotzingo, Puebla	B ^3^
6	Belgian Sheperd	Female	2.5	Cuajinicuilapa, Guerrero	B ^3^
7	Mixed-breed	Female	4	Huejotzingo, Puebla	B ^3^
8	Mixed-breed	Female	4	Huejotzingo, Puebla	B ^3^

^1^ Transmissible venereal tumor. ^2^ Pedunculated strawberry-like. ^3^ Multilobulated cauliflower-like.

**Table 2:** Primary and secondary antibodies used for cellular type characterization.

Primary Antibody (Dilution)	Cell Type Detected	Clone nº	Source	Secondary Antibody (Dilution)
IBA1 ^1^ (1:1000)	Activated macrophages	Polyclonal 019-19741	FLUJIFILM-Wako Chemicals Europe GmbH, Neuss, Germany	Goat anti-rabbit (1:200)
iNOS ^2^ (1:200)	M1 macrophages	Polyclonal PA1-036	ThermoFisher, Waltham, MA, USA	Goat anti-rabbit (1:200)
CD163 (1:2000)	M2 macrophages	Polyclonal PA5-83817	ThermoFisher, Waltham, MA, USA	Goat anti-rabbit (1:200)
CD3 (1:500)	T lymphocytes	Monoclonal NCL-L-CD3-565	Novacastra, Leica Biosystem, Newcastle, UK	Horse anti-mouse (1:200)
CD20 (1:400)	B lymphocytes	Polyclonal PA5-16701	ThermoFisher, Waltham, MA, USA	Goat anti-rabbit (1:200)
Lambda light chain (1:1000)	Plasma cells	Polyclonal PA5-16651	ThermoFisher, Waltham, MA, USA	Goat anti-rabbit (1:200)

^1^ Ionized calcium-binding adapter molecule 1. ^2^ Inducible nitric oxide synthase.

**Table 3:** Results of the generalized linear mixed model assessing the effects of tumor morphopathology (strawberry-like Type A or cauliflower-like Type B), cell marker (IBA1, iNOS, CD163, CD3, CD20, and lambda light chain), and their interaction on marker expression (n = 240 fields randomly selected). Individual dogs were included in the model as a random factor. Reference categories were cauliflower-like Type B for tumor morphology and CD163 (M2 macrophages) for cell markers. Significant effects are highlighted in bold type (*p* < 0.05).

Variables	Estimate ± Standard Error	*p*
Tumor morphopathology: Type A	−0.11 ± 1.23	0.93
Cell marker: IBA1 (activated macrophages)	0.32 ± 0.98	0.74
Cell marker: iNOS (M1 macrophages)	0.04 ± 0.98	0.97
Cell marker: CD3 (T lymphocytes)	−0.19 ± 0.98	0.85
Cell marker: CD20 (B lymphocytes)	1.49 ± 0.98	0.13
Cell marker: lambda light chain (plasma cells)	1.14 ± 0.98	0.25
Cell marker*Tumor morphopathology: IBA1*Type A	14.88 ± 1.38	**<0.001**
Cell marker*Tumor morphopathology: iNOS*Type A	0.11 ± 1.38	0.94
Cell marker*Tumor morphopathology: CD3*Type A	0.09 ± 1.38	0.95
Cell marker*Tumor morphopathology: CD20*Type A	3.35 ± 1.38	**0.02**
Cell marker*Tumor morphopathology: Lambda*Type A	−0.63 ± 1.38	0.65

“*” represents the interaction between variables.

## Data Availability

All data are included in the manuscript.

## Abbreviations

The following abbreviations are used in this manuscript:

TVT	Canine transmissible venereal tumor
IBA1	Ionized calcium-binding adaptor molecule 1
iNOS	Inducible nitric oxide synthase
MHC	Major histocompatibility complex
TBS+BSA	Tris-buffered saline with bovine serum albumin
AIF-1	Allograft inflammatory factor 1
